# A Hierarchical Machine Learning Model to Discover Gleason Grade-Specific Biomarkers in Prostate Cancer

**DOI:** 10.3390/diagnostics9040219

**Published:** 2019-12-11

**Authors:** Osama Hamzeh, Abedalrhman Alkhateeb, Julia Zhuoran Zheng, Srinath Kandalam, Crystal Leung, Govindaraja Atikukke, Dora Cavallo-Medved, Nallasivam Palanisamy, Luis Rueda

**Affiliations:** 1 School of Computer Science, University of Windsor, 401 Sunset Ave, Windsor, ON N9B 3P4, Canada; hamzeho@uwindsor.ca (O.H.); zheng12z@uwindsor.ca (J.Z.Z.); 2 Department of Biomedical Sciences, University of Windsor, 401 Sunset Ave, Windsor, ON N9B 3P4, Canada; kandala1@uwindsor.ca (S.K.); dcavallo@uwindsor.ca (D.C.-M.); 3 Schulich School of Medicine and Dentistry, Western University, 1151 Richmond St, London, ON N6A 5C1, Canada; cleung2021@meds.uwo.ca; 4 ITOS Oncology Inc., 1453 Prince Rd, Ste: 4125, Windsor, ON N9C 3Z4, Canada; gatikukke@itosoncology.com; 5 Department of Urology, Henry Ford Health System, One Ford Place, Detroit, MI 48202, USA

**Keywords:** supervised learning, next generation sequencing, classification, transcriptomics, Gleason score detection, prostate cancer

## Abstract

(1) Background:One of the most common cancers that affect North American men and men worldwide is prostate cancer. The Gleason score is a pathological grading system to examine the potential aggressiveness of the disease in the prostate tissue. Advancements in computing and next-generation sequencing technology now allow us to study the genomic profiles of patients in association with their different Gleason scores more accurately and effectively. (2) Methods: In this study, we used a novel machine learning method to analyse gene expression of prostate tumours with different Gleason scores, and identify potential genetic biomarkers for each Gleason group. We obtained a publicly-available RNA-Seq dataset of a cohort of 104 prostate cancer patients from the National Center for Biotechnology Information’s (NCBI) Gene Expression Omnibus (GEO) repository, and categorised patients based on their Gleason scores to create a hierarchy of disease progression. A hierarchical model with standard classifiers in different Gleason groups, also known as *nodes*, was developed to identify and predict nodes based on their mRNA or gene expression. In each node, patient samples were analysed via class imbalance and hybrid feature selection techniques to build the prediction model. The outcome from analysis of each node was a set of genes that could differentiate each Gleason group from the remaining groups. To validate the proposed method, the set of identified genes were used to classify a second dataset of 499 prostate cancer patients collected from cBioportal. (3) Results: The overall accuracy of applying this novel method to the first dataset was 93.3%; the method was further validated to have 87% accuracy using the second dataset. This method also identified genes that were not previously reported as potential biomarkers for specific Gleason groups. In particular, *PIAS3* was identified as a potential biomarker for Gleason score 4 + 3 = 7, and *UBE2V2* for Gleason score 6. (4) Insight: Previous reports show that the genes predicted by this newly proposed method strongly correlate with prostate cancer development and progression. Furthermore, pathway analysis shows that both *PIAS3* and *UBE2V2* share similar protein interaction pathways, the JAK/STAT signaling process.

## 1. Introduction

Cancer is among the main causes of death worldwide. Among males, prostate cancer is the cancer type with the highest incidence; 1.276 million new cases were diagnosed in 2019 [1]. To date, most cancer studies have concentrated on finding biomarkers that enable differentiating malignant tumours from benign ones. More recent studies, though, have focused on specific clinical aspects of tumours, such as recurrence, progression, survivability, and metastasis, among others.

In the 1950s, Pierre Denoix devised a system that categorises solid tumours into different stages [2]. The classification (TNM) of cancer progression is done by utilising (T) the extension and the size of the main tumour, (N) the lymphatic involvement, and (M) the metastasis levels [3]. In prostate cancer, these characteristics are also used to assign a metric of tissue organisation and disease aggressiveness called the Gleason score. That score is calculated by adding two numbers: the most common pattern of the tumour cells is used as the first number, while the second number corresponds to the next most common pattern. Each individual score varies from 3 to 5, depending on the aggressiveness of the tumour, where the highest score means the most aggressive form of cancer [4]. Epstein et al., however, indicated that Scores 2–5 are no longer assigned to the tissue and these multiple scores can be categorised together with score 6 as group 1, yielding categories as depicted in Table 1. They are used to determine prognosis of disease. As such, we have used it as the main scheme for prostate cancer score categorization in our method to detect transcriptomic biomarkers that can accurately classify specific Gleason scores and groups. This categorization strategy has been shown to clearly indicate cancer recurrence, and improve the prognostic role of the Gleason score [5].

Recent prostate cancer research has greatly focused on identifying gene expression patterns that correlate with disease progression, and can be used as predictive tools for patient treatment and outcome. Moreover, advances in next-generation sequencing (NGS) technology have made genomic data analysis widely available. The output of NGS sequencers requires preprocessing algorithms to do things such as align the reads to a reference human genome and assemble them into transcripts. Many genomic tools that align the RNA-Seq reads to the human genome have been proposed, especially BLAST is one of the first tools developed to align reads [6]. TopHat2 is a widely used, open-source tool that incorporates Bowtie sequence alignment to align reads [7]. STAR is the fastest RNA-Seq sequence alignment algorithm to date, although it requires huge computational resources to perform efficiently [8]. Based on the need for understanding the biological basis of the visual Gleason microscopic assessment, Roberto et al. conducted a gene expression profiling on two groups of Gleason score 6 and 7, or high, using a metabolic gene panel. The panel consists of many gene members of the JAK/STAT pathway [9]. In this study, we analysed the transcription level of different Gleason scores to find genes that can identify one specific Gleason group from the others.

In addition, machine learning applications in genomic analysis have become a solid approach to analysing RNA-Seq data for studying a multitude of diseases. Alkhateeb et al. proposed a supervised method to discover biomarkers that can predict the likelihood that a prostate cancer tumour will progress to the next stage [10]. Arvaniti et al. proposed a deep learning approach to predict Gleason scores [11]. Their model was trained using tissue microarray (TMA) images of 641 patients with varying Gleason scores, and validated using 245 patient samples with Gleason scores that were reviewed by pathologists. Although the study by Arvaniti et al. reported decent performance measurements (average accuracy 85.72%, and recall 0.57%), it did not report the panel of biomarker genes that were used by the trained convolutional neural network (CNN) to predict Gleason scores. Citak-Er et al. proposed a machine learning approach for predicting Gleason scores [12]. Their method uses a support vector machine (SVM) on prostate images to learn the visual attributes of the disease and to predict the disease outcome. That study was conducted on a limited cohort of prostate cancer patients, and the results showed a higher sensitivity over the specificity in the prediction model (accuracy = 76.83%; sensitivity = 83.38%; specificity = 68.36%).

The focus of this study was to identify genes that can be used to differentiate specific Gleason groups. This work is an extension of our previously proposed prediction model, which was based on analysing the RNA-Seq data from patients with different Gleason scores [13]. The method can track transcripts associated with specific genes, in addition to their corresponding expression values. The results of the initial trial show great potential to build a simple system to diagnose Gleason scores based on NGS data.

## 2. Results

**Figure 1:**
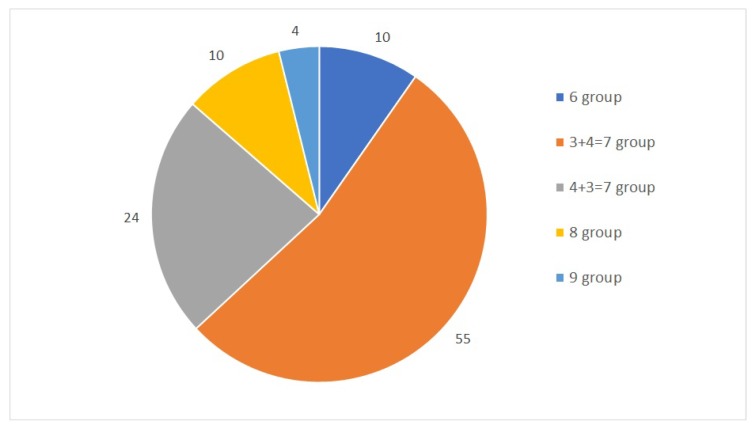
Gleason groups and their distributions.

The first dataset used in this study is a collection of 104 samples and their TPM values. Stated as a classification problem, this study designates five classes obtained from joint Gleason groups. The distribution of each group is shown in Figure 1. The dataset was mapped against the human genome version hg19 with 88% to 99% uniquely aligned reads. Throughout a 10-fold cross-validation model, we obtained a total of seven samples that were misclassified and another 97 samples that were classified correctly, with the total number of samples being 104. The accuracy of the model was calculated from the total number of correctly classified samples divided by the total number of samples.

**Figure 2:**
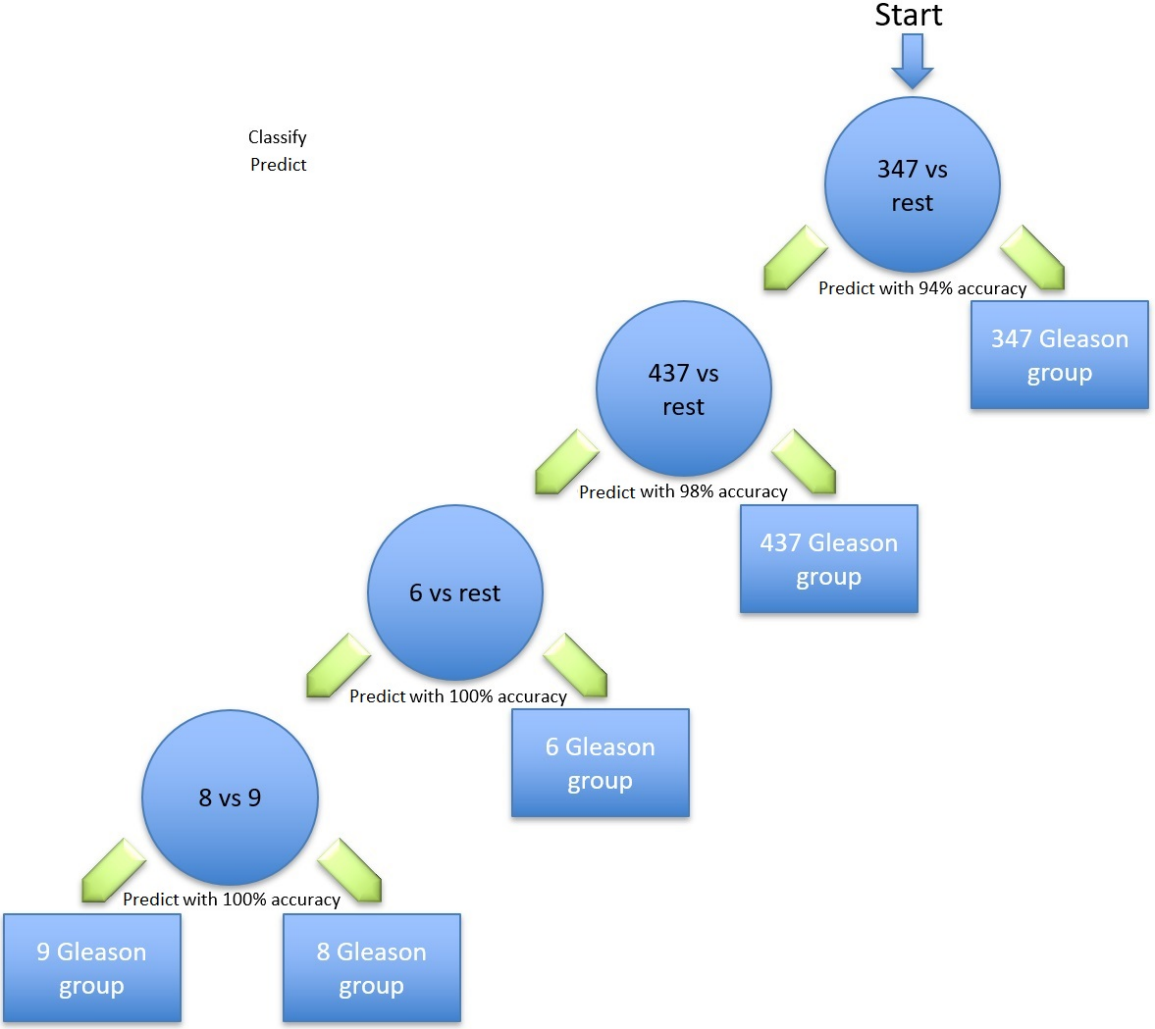
Hierarchical tree of classifications of Gleason groups against the rest, along with the corresponding classification accuracies.

The model also identified six gene transcripts that are differentially expressed in the five different Gleason scores. Of these, the corresponding genes shown in Table 2, Table 3, Table 4 and Table 5 are the most relevant for identifying prostate cancer; the Gleason scores using the hierarchical method are illustrated in Figure 2. Different classification methods for each stage within the hierarchy are shown in Table 6. The first node of the hierarchy yields 94% accuracy in identifying Gleason score 3 + 4 = 7 compared to the other scores. The samples are then passed through node 2, in which Gleason score 4 + 3 = 7 was identified from the rest with a prediction accuracy of 98%. The other samples were then passed through node 3, where Gleason score 6 was identified with 100% accuracy. The remaining samples were finally processed in the last node, where the Gleason score 8 was identified from the Gleason score 9 with 100% accuracy. Due to the similarity in the aggressiveness of the tumour and the low number of samples, all the other Gleason scores were merged in the last node.

**Figure 3:**
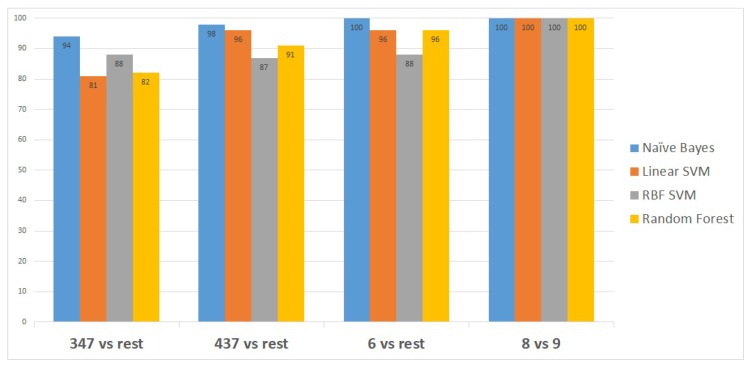
Accuracy obtained by each classifier for classifying one versus the rest for all five Gleason groups.

Figure 3 shows the classifiers that have been utilised to identify the set of transcripts that differentiate specific Gleason groups against the rest. The classifiers are represented on the x-axis, while the classification performance measurements are represented on the y-axis.

Naïve Bayes outperformed the other classifiers, as it distinguished the first Gleason score node from the rest with 94% accuracy, the second node with a higher accuracy of 98%, and the last two Gleason score nodes with 100% accuracy, as shown in Figure 3.

**Figure 4:**
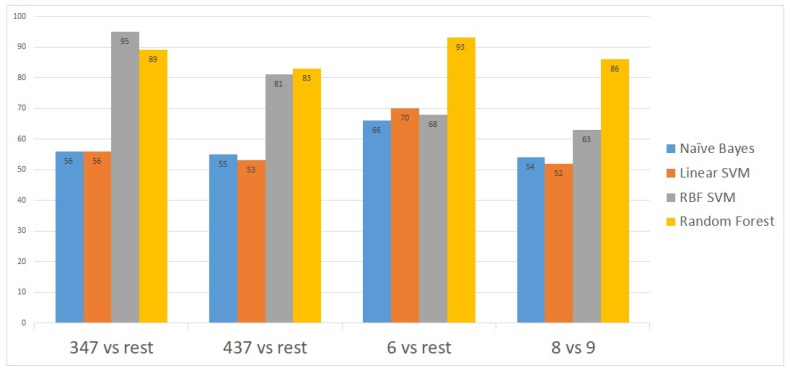
Classification accuracies obtained after applying the model on the second dataset.

To further validate the model, we applied the method on a second publicly-available dataset [14] obtained from the National Center for Biotechnology Information (NCBI) portal [15]. This second dataset contains gene expressions for 498 patient samples. The proposed model showed an excellent prediction accuracy on the 498 patients’ gene expressions. The prediction accuracy for all the Gleason scores was above 90% except for the 4 + 3 = 7 Gleason score versus the rest (Figure 4).

## 3. Discussion

Many of the genes that encode the differentially expressed transcripts identified in this study have been previously shown to play various roles in cancer. Some have been shown to promote cancer progression, while other play a protective role. For example, *UBE2V2*, whose gene’s transcript was selected in the third node of our hierarchical model, has been shown to protect cells by mediating DNA repair functions [16]. In familial prostate cancer, however, a high frequency variant of UBE2V2 was identified and found to affect DNA repair and androgen signaling [17]. In our model study, a different quantification of the UBE2V2 transcript was able to predict Gleason score 6 (group 1) in the first dataset. Differential expression of UBE2V2 has also been associated with poor prognosis in breast cancer [18].

Our study also revealed that the differential expression of *GPR137* expression and EPB41L1 is associated with tumours of Gleason scores 3 + 4 = 7 and 8, respectively. Earlier studies show that proteins encoded by *EPB41L1* are associated with the proper organisation of the cell cytoskeleton, and that *EPB41L1* plays an important role in the negative regulation of cell metastasis, migration, and invasion. Expression of *EPB41L1* has been observed to be lower in prostate cancer compared to normal cells. Although it remains unclear, disruption of normal *EPB41L1* expression may play an important role in disorganised cell and tissue structures associated with higher grade prostate cancer [19], and thus link its deregulation to prostate cancer progression and prognosis. Furthermore, reduced expression of *EPB41L1* plays an important role in recurrence and has been associated with highly metastatic lung and breast cancer [20]. *EPB41L1* was also shown to be differentially expressed in gastric cancer [21]. On the other hand, *GPR137* expression has been shown to be upregulated in prostate cancer tissues compared with paracancerous tissues. Moreover, knockdown of *GPR137* resulted in decreased cell proliferation and colony formation in PC-3 and DU145 prostate cancer cell lines, and was associated with cell cycle arrest at G0/G1 phase. *GPR137* suppression also decreases the migration and invasive abilities of PC-3 cells, suggesting that *GPR137* plays a role in prostate cancer progression and metastasis [22].

Differential expression of *PIAS3* and Rest Corepressor 3 (Rcor3) were both associated with tumours of Gleason score 4 + 3 = 7. While very little is known about the role of Rest Corepressor 3 (Rcor3) in prostate cancer, it has been shown to act as an antagonist of cell differentiation [23], a characteristic of prostate tumours with Gleason score 4 + 3 = 7 [4]. On the other hand, differential *PIAS3* expression has been observed in a variety of human cancers, including lung, breast, prostate, colorectal, and brain [24]. *PIAS3* is expressed in prostate cancer cells, and its expression is induced in response to androgens [25,26]. Although *PIAS* has been shown to enhance the transcriptional activity of androgen receptors (AR) in prostate cancer cells, other studies have revealed that ectopic overexpression of *PIAS3* suppresses AR-mediated gene activation induced by dihydrotestosterone (DHT) [24]. *PIAS3* acts as a negative regulator of AR transcriptional activity and signaling through direct protein–protein interaction. Recent findings have also revealed that AR is also differentially correlated with Gleason score patterns in both primary and metastatic prostate cancer, where it is upregulated in Gleason group 4 and downregulated in Gleason pattern 5.

**Figure 5:**
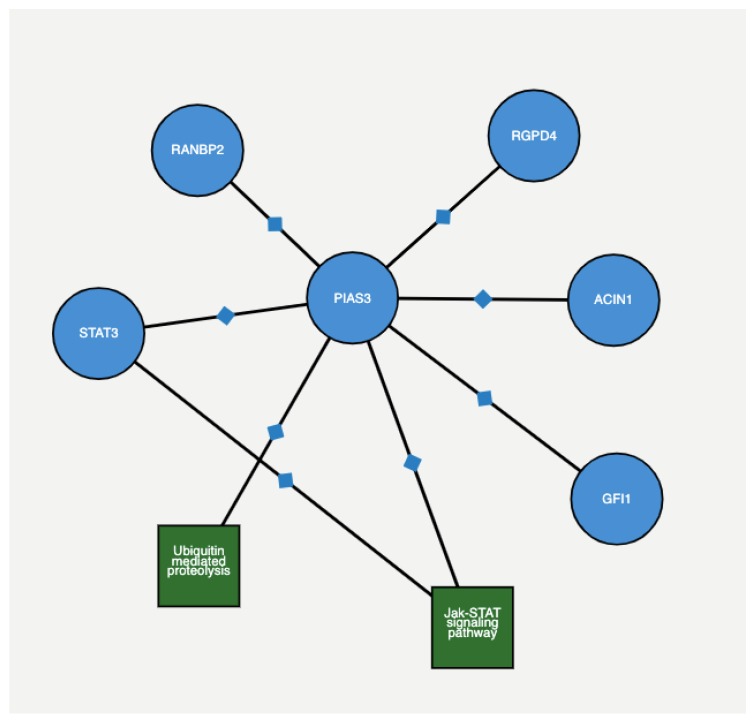
An interactive figure taken from proteomics database STRING. It shows neighbouring protein binding and pathway interactions for a given gene using STRING and KEGG pathway analysis. Here, the gene of interest is *PIAS3*, an identified possible biomarker in the 4 + 3 = 7 score. The figure shows the interaction between other proteins and pathways associated with it.

*PIAS3* is a member of the mammalian *PIAS* family consisting of four members: *PIAS1*, *PIAS2*, *PIAS3*, and *PIAS4* [27]. *PIAS3* protein directly binds to several transcription factors and either blocks or enhances their activity. *PIAS3* is also specific inhibitor of signal transducer and activator of transcription 3 (STAT3), a transcription factor and member of the Janus kinase (JAK)/STAT signaling pathway [28,29]. This signaling pathway has been a target of interest in many cancer studies in recent years. In prostate cancer, the expression levels of JAK/STAT have been shown to impact the progression of the disease [30,31]. As an inhibitor of STAT3, *PIAS3* blocks the transactivation and binding of STAT3 to specific DNA elements via protein–protein interactions, thereby inhibiting STAT3-mediated gene activation. Figure 5 depicts the protein–protein interaction among genes with 4 + 3 = 7 and 6 scores, as extracted from ProteomicsDB (https://www.proteomicsdb.org/proteomicsdb/#human/proteinDetails/86810/interactions) based on experimental and epidemiological evidence. The Figure shows that both *PIAS3* and *UBE2V2* share the same protein interaction network.

*PIAS3* is also the only member of the *PIAS* family that has been shown to directly interact with Stat5a/b and repress Stat5-mediated transcription [32]. Stat5a/b is constantly active in human prostate cancer [33], associated with high histological grades [34], and a predictor of early prostate cancer recurrence [35]. Transcription factor Stat5a/b has been shown to regulate the viability and growth of human prostate cancer cells [36,37]. Moreover, in vitro inhibition of Stat5a/b induces apoptosis in human prostate cancer cells [33,38]. In vivo, Stat5a/b inhibition blocks prostate cancer subcutaneous and orthotopic xenograft tumour growth in nude mice [38]. Although studies have revealed an inhibitory role for *PIAS3* against Stat5a/b-driven gene transcription and disease progression in breast cancer, the predominant Stat5a/b protein that binds to DNA has been shown to be N-terminally truncated in human prostate cancer cells and clinical prostate cancers [39]. Further studies have demonstrated that the N-domain of Stat5a/b binds to *PIAS3*. Hence, the truncated form of Stat5 in prostate cancer cells evades *PIAS3*-mediated transcriptional inhibition, thereby increasing prostate cancer growth and progression. Thus, the proteolytic cleavage of the N-terminus of Stat5a/b may be a mechanism by which Stat5 evades the transcriptional repression by *PIAS3* in prostate cancer cells. This further indicates the complexity of intracellular protein interactions and its role in disease progression.

Our study applied a novel machine learning model to identify differentially expressed, prostate cancer stage-specific transcripts. Although the application of this model to other related datasets is required to further valid our findings, the use of this model in conjunction with in vitro and in vivo biological studies will aid in elucidating the intricate molecular relationships between the identified transcripts. Moreover, this will provide more insight into predicted prognostic outcomes and the development of effective therapeutic strategies against prostate cancer progression.

## 4. Materials and Methods

The primary dataset used in this study was retrieved from the National Center for Biotechnology Information (NCBI) and is referenced with Gene Expression Omnibus (GEO) number GSE54460 [40]. This RNAseq prostatectomy dataset was generated from 106 prostate cancer tissue samples and validated on an independent dataset with 140 patients. Several health sciences centres provided data samples as well. The Moffitt Cancer Center (MCC) contributed ten samples from patients who underwent radical prostatectomies between the years 1987 and 2003. The Sunnybrook Health Sciences Centre at the University of Toronto provided 35 samples from patients treated for prostate cancer between the years 1998 and 2006. The Atlanta Veterans Administration Medical Center (AVAMC) donated 61 tissue samples from patients who underwent radical prostatectomy between the years 1990 and 2000. Table 7 shows the number of samples grouped by their Gleason group. Based on Epstein’s model, there are five Gleason groups: 4 + 3 = 7, 3 + 4 = 7, 6, 8, and above 8 (9 and 10).

**Figure 6:**
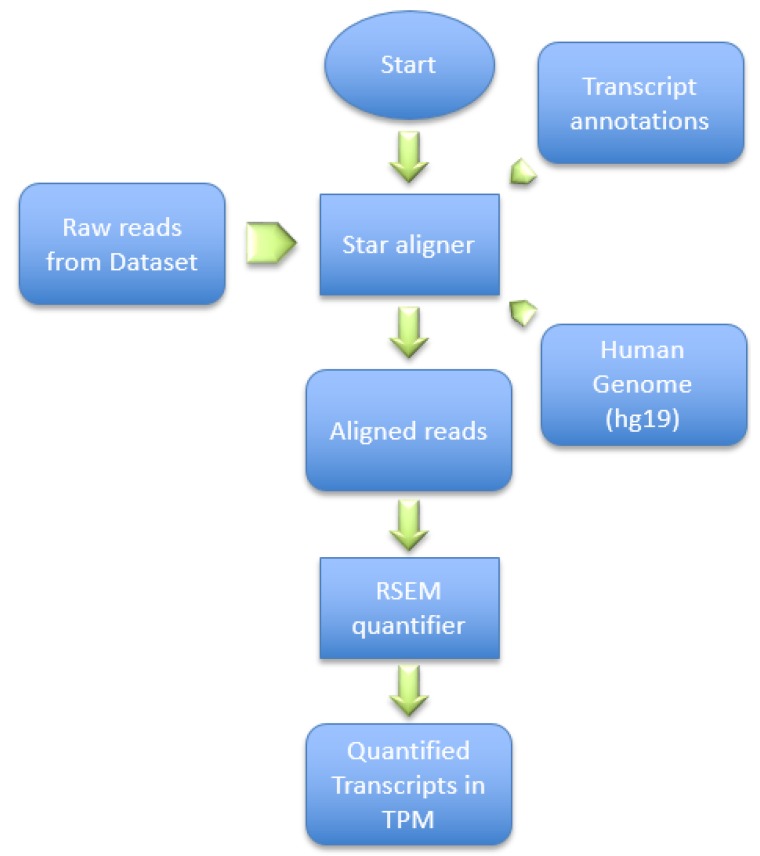
Pre-processing steps of the proposed method.

This dataset was generated by using the Illumina HiSeq 2000 NGS on paired-end sequences of length 51 bp each. The pre-processing pipeline model starts by obtaining the RNA-Seq samples and pre-processing it using SRAtools [41], as depicted in Figure 6. The process continues by incorporating the STAR aligner [8] to align the samples reads into the human genome (hg19). Then, the process assembles the transcripts and quantifies the reads into the assembled transcripts using RSEM [42]. RSEM uses transcripts per million of reads (TPM) to compute the quantification of each read into a transcript.

NGS technology allows us to read the patient’s genome and generate a significant amount of raw data in a snapshot. However, the underlying process yields artefacts, and pre-processing must be done before the downstream analysis. These artefacts include duplication and bias reads [43], among others. Counting the reads that are assembled by mapping them to the human genome gives accurate indicators of transcript expression. Since the samples are pair-ended reads, TPM is used to measure the read quantification rather than reads per kilobase per million of reads (RPKM) [44]. Additionally, the reason for choosing TPM instead of fragments per kilobase per million (FPKM) [45] is that TPM normalises the reads to the length of the gene first, which makes it easier to compare the quantified reads among different samples.

### 4.1. Class Imbalance

Some classes have a markedly lower number of samples than the others, which may cause some classifiers to become biased towards the majority class. To solve this problem, multiple resampling methods were deployed and tested to identify the specific method that would yield the best solution for a particular dataset. After applying multiple oversampling and under-sampling methods, the best option was found to be the synthetic minority oversampling technique (SMOTE) [46] for oversampling the minority class, while the neighbourhood cleaning rule (NCL) [47] was used for undersampling the majority class.

**Figure 7:**
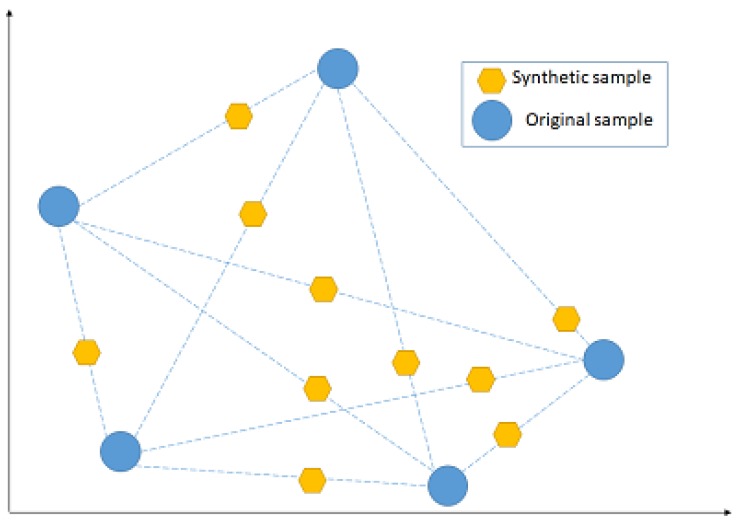
Hypothetical example that shows how the synthetic minority oversampling technique (SMOTE) works.

NCL works by removing any sample whose class is different from the class of at least two of its three nearest neighbours. SMOTE, instead, introduces a new way of creating new samples, by utilising the feature vector that connects each sample and introduces a new synthetic sample along the line that connects the two underlying samples. The exact location of the new sample on the line itself is calculated by measuring the Euclidean distance between the two samples and multiplying that value by a random number between 0 and 1. Figure 7 shows a hypothetical example of the mechanism followed by SMOTE, by adding new synthetic samples randomly along the line that connects each of two original samples in a minority class. The blue points represent the original samples, while the amber points represent the synthetically generated samples.

### 4.2. Feature Selection

As the output of the pre-processing step, the method retrieved 41,971 transcripts along with their corresponding quantifications measured by TPM. Such a large number of transcripts leads to a complex classification model, mostly due to the curse of dimensionality [48]. Thus, feature selection was applied to reduce the dimensionality of the problem. The first step of the feature selection step is to filter the transcripts based on their information gain values by selecting the ones with the highest scores. The filter method, which is called attribute evaluator, is the procedure by which each attribute (transcript) in the dataset is assessed with regard to the class. This procedure produces a list of attributes (transcripts) with a score for each attribute showing its effect on the actual class. Then, the attributes with the highest scores are selected, discarding those with lower scores. In this work, information gain (IG) was used as an attribute evaluator to rank each attribute vector [49]. The IG of attribute vector *X* concerning class vector *A* is defined as follows:

(1) IG(A,X)=H(A)−H(A|X),

where

(2) $$H\left(A\right)=-\sum_{a\in A}p\left(a\right){log}_{2}\left(p\left(y\right)\right),$$

and

(3) $$H\left(A|X\right)=-\sum_{x\in X}p\left(x\right)\sum_{a\in Y}p\left(a|x\right){log}_{2}\left(p\left(ax\right)\right).$$

Here, H(A) is the entropy of the class vector *A* and H(A|X) is the conditional entropy of *A* given *X*.

After filtering the transcripts based on their IG scores, a wrapper-based feature selection algorithm that uses minimum redundancy maximum relevance (mRMR) is used to narrow down the most relevant, least redundant transcripts to a few per group; mRMR has the capability of incorporating any classifier to select features (transcripts) that minimise the redundancy while increasing the correlation to the class vector [50]. The wrapper method adds up the features that minimise redundancy ($W_{i}$), and maximise the relevance ($V_{i}$), with the best possible accuracy of an SVM classifier that uses a linear kernel, as per the following equations:

(4) $$W_{i}=\frac{1}{|S{|}^{2}}\;\sum_{i,j\in S}I\left(i,j\right)\;,$$

and

(5) $$V_{i}=\frac{1}{|S|}\;\sum_{i\in S}I\left(h,i\right)\;,$$

where *S* is the set of features, I(i,j) is the mutual information between features (i,j), and *h* is the class, in our case, the five Gleason groups.

### 4.3. Classification

The problem dealt with is multi-class classification, which was solved using the one-versus-rest approach. There are five different classes, which correspond to the five distinct Gleason groups. To apply a one-versus-rest approach, we created five different datasets from the actual data. For each dataset, we set one of the classes to form the positive class, while the rest of the classes were combined to form the negative class. The classification pipeline resembles a binary tree structure, where each internal node is a binary classification problem (see Figure 2). Starting from the root, in the one-versus-rest classification, we remove the samples that belong to the chosen class earlier. We repeat the same steps of building datasets for the remaining four different classes. At each node, the best class is chosen and the classification continues in the same fashion until two classes are left. To select the best class at each node, different performance measures can be used; accuracy, sensitivity, and specificity are used here. Note that the hierarchical model involves list processing, and as such, any error at a particular node is propagated down the tree structure. In a greedy-like algorithm, we minimise the error propagation by choosing the class with the highest accuracy at each internal node.

### 4.4. Identifying Transcripts within Different Gleason Scores

**Figure 8:**
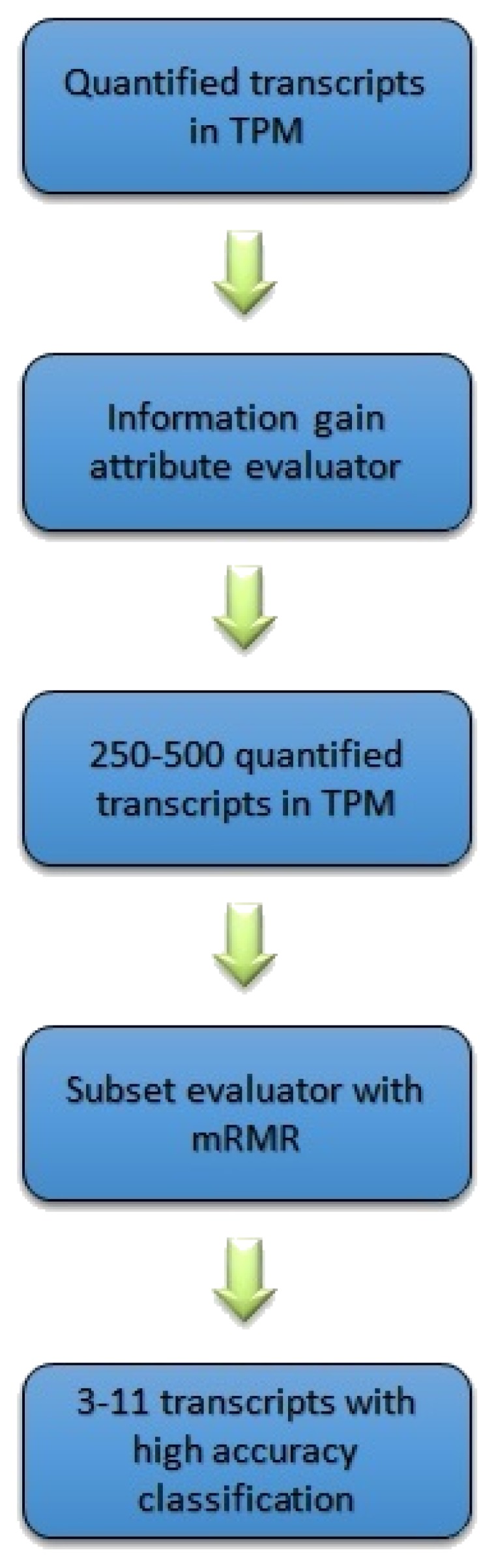
Machine learning pipeline used in the proposed method.

We used the Scitkit-learn [51] library to apply different classification algorithms to the final transcripts selected. This step identifies which transcripts can decide a Gleason group from the others based on their quantification values. Standard classifiers such as Naïve Bayes and SVM were used in this study to build the classification model. Naive Bayes is a probability-based classifier that applies the well-known Bayes’ theorem, while assuming that the features are independent of each other [52]. While being simple, Naïve Bayes has been shown to perform very well in many problems and avoid overfitting. An SVM classifier was also used to build a prediction model using the transcripts selected in the previous step [53]. The advantage of SVM is its exceptional generalisation power, especially in high-dimensional data with a small number of samples. Figure 8 shows the pipeline followed in this study.

## 5. Conclusions and Future Directions

Identifying novel biomarkers that are clinically associated with specific Gleason groups in prostate cancer is vital for the diagnosis and treatment of the disease. Utilising NGS data and machine learning techniques, a supervised learning method was proposed to find group-specific sets of transcripts with significant different levels of quantification values. The transcripts, along with the corresponding genes, identified by the proposed machine learning method, were found in the literature to play crucial roles in cancer pathogenesis; key transcripts were strongly correlated to prostate cancer. To validate the model, we also tested it on a gene expression dataset, showing that the resulting genes are related to prostate cancer progression.

The work presented in this paper opens the way for future directions of research. One of these is to apply and adjust the same method to other cancer types. Another possible avenue would be to consider analysing samples from patients who have progressed through more than one Gleason group. This method aims to eliminate confounding factors between patients, potentially leading to a clearer analysis of differential gene expression between different grades of prostate cancer. In addition, a multi-omics model based on different types of genomics data for this problem could be investigated, which may provide a comprehensive analysis of the progression, diagnosis, and treatment of the disease.

## Figures and Tables

**Table 1:** Gleason groups considered in this study.

Gleason Group	Score
1	6
2	3 + 4 = 7
3	4 + 3 = 7
4	8
5	9 and 10

**Table 2:** Set of resulting transcripts in Gleason group 1.

Transcript	Gene	Description
NM_003350	*UBE2V2*	ubiquitin conjugating enzyme E2 V2 (*UBE2V2*)
NM_153051	*MTMR3*	myotubularin related protein 3 (*MTMR3*), transcript variant 2
NM_207445	*C15orf54*	chromosome 15 open reading frame 54 (*C15orf54*)

**Table 3:** Set of resulting transcripts in Gleason group 2.

Transcript	Gene	Description
NM_001170880	*GPR137*	G protein-coupled receptor 137 (*GPR137*), transcript variant 2
NM_001198827	*C8orf58*	chromosome 8 open reading frame 58 (*C8orf58*), transcript variant 3
NM_004629	*9p13.3*	Fanconi anemia complementation group G (*FANCG*)
NM_001098268	*LIG4S*	DNA ligase 4 (*LIG4*), transcript variant 3
NM_016641	*GDE1*	glycerophosphodiester phosphodiesterase 1 (*GDE1*), transcript variant 1
NM_002445	*MSR1*	macrophage scavenger receptor 1 (*MSR1*), transcript variant SR-AII
NM_001126337	*TUFT1*	tuftelin 1 (*TUFT1*), transcript variant 2
NM_033071	*SYNE1*	spectrin repeat containing nuclear envelope protein 1(*SYNE1*), transcript variant 2
NM_052906	*ELFN2*	extracellular leucine rich repeat and fibronectin typeIII domain containing 2 (*ELFN2*), transcript variant 1
NM_000714	*TSPO*	translocator protein (*TSPO*), transcript variant PBR
NM_004374	*COX6C*	cytochrome c oxidase subunit 6C (*COX6C*)
NM_001007544	*C1orf186*	chromosome 1 open reading frame 186 (*C1orf186*)
NM_001276438	*KCNJ15*	potassium voltage-gated channel subfamily J member 15 (*KCNJ15*), transcript variant 7
NM_001252021	*TOR2A*	torsin family 2 member A (*TOR2A*), transcript variant 7
NM_152612	*CCDC116*	coiled-coil domain containing 116 (*CCDC116*), transcript variant 1

**Table 4:** Set of resulting transcripts in Gleason group 3.

Transcript	Gene	Description
NM_001136224	*RCOR3*	REST corepressor 3 (*RCOR3*), transcript variant 2
NM_001017967	*MARVELD3*	MARVEL domain containing 3 (*MARVELD3*), transcript variant 1
NM_006099	*PIAS3*	protein inhibitor of activated STAT 3 (*PIAS3*)
NM_152395	*NUDT16*	nudix hydrolase 16 (*NUDT16*), transcript variant 2
NM_006473	*TAF6L*	TATA-box binding protein associated factor 6 like (*TAF6L*)
NM_001145541	*TCP11L1*	t-complex 11 like 1 (*TCP11L1*), transcript variant 2
NM_182501	*MTERF4*	mitochondrial transcription termination factor 4 (*MTERF4*)

**Table 5:** Set of resulting transcripts in Gleason group 4.

Transcript	Gene	Description
NM_001258330	*EPB41L1*	erythrocyte membrane protein band 4.1 like 1 (*EPB41L1*), transcript variant 4

**Table 6:** Classification performance for each step in the hierarchy.

Gleason Group	Accuracy	Sensitivity	Specificity	F-Measure	MCC	ROC Area
3 + 4 = 7 vs. Res	94	95	94	0.94	0.88	95
4 + 3 = 7 vs. Rest	98	100	96	0.98	0.96	99
6 vs. Rest	100	100	100	1.00	1.00	100
8 vs. 9	100	100	100	1.00	1.00	100

**Table 7:** Numbers of samples in different Gleason groups.

Gleason Score	Number of Samples
6	10
3 + 4 = 7	55
4 + 3 = 7	24
8	10
9	4

## Abbreviations

The following abbreviations are used in this manuscript:

NGS	Next-generation sequencing
SVM	Support vector machine
mRMR	Minimum redundancy maximum relevance
IG	Information Gain
RPKM	reads per kilobase per million of reads
FPKM	Fragments per kilobase per million of reads
TPM	Transcripts per million of reads
